# 

**Published:** 1894-01-20

**Authors:** 


**Notice to Advertisers and Subscribers.**

All Advertisements, Orders for Copies of The Hospital, and Business Communications should be addressed to The Publisher, *not to the Editor*, The Hospital, 428, Strand, W.C.

The Cost of Subscription, post free, is as follows:—Per week, 2½d.; per quarter, 2s. 8d.; per half-year, 5s. 3d.; per annum, 10s. 6d Foreign:—Per Annum, 12s. 8d.; payable, in all cases, in advance.

**OUR NEW OFFICES.**

428, Strand, W.C., will henceforth be the Office of this Journal.

**Notices of Births, Deaths, and Marriages** are inserted in this column at a charge of 2s. 6d. for an announcement not exceeding four lines.

### NOTICE TO CORRESPONDENTS.

All MS., Letters, Books for Review, and other matters intended for the Editor should be addressed The Editor, The Lodge, Porchester Square, London, W.

The Editor cannot undertake to return rejected MS., even when accompanied by stamped directed envelope.

**"Burdett's Hospital Annual," 1893.**

Fourth year of publication. 700 pp. Boards, gilt lettering. A most complete guide to British, Colonial, Indian, and American Hospitals, Dispensaries, Nursing and Convalescent Institutions, and Asylums. Price 5s. Published by The Scientific Press (Limited), 428, Strand, W.C.

**NOTICE.—The Index for Vol. XIV. (ending Sept. 30th, 1893) is on sale at the Office of this Journal, price 2½d., post free.**

### SCRAPS AND GLEANINGS.

THE Northampton Hospital Week Fund Committee has handed over to the funds of that institution the sum of £200.

THE new north wing of the Staffordshire General Infirmary at Stafford was recently thrown open on its completion to the inspection of the public.

MR. GRAHAM VIVIAN has sent £14 15s. to the Swansea Hospital for the purpose of supplying the serious want of an operating table for that institution.

DURING the past year London drew no less than five and a-half millions for charity, about £800,000 being devoted to contributions to hospitals and dispensaries.

THE authorities of the Bristol Dispensary have arranged to send a few convalescent patients requiring a change to the Sanatorium Home at Weston-super-Mare.

THE total amount raised so far in connection with the monument to be erected in Paris to the memory of the late Dr. J. M. Charcot has reached the sum of 197,505 francs.

A DEPUTATION to protest against the site selected for the Epsom and Sutton Joint Hospital recently waited on a meeting held by the members of the combined Infectious Diseases Hospital Board.

THE latest absurdity of the Russian craze in Paris is the gift of a French woman calling herself "A Friend of the People," who has sent 3,000 smelling bottles to the women's hospitals in Paris.

THE Royal Hospital for Diseases of the Chest, City Road, E.C., has just received a donation of £500 from a lady who takes a keen interest in the work of the institution, but does not wish her name to be known.

A MEETING of the joint committee of members of the Redcar, Kirkleatham, and Saltburn local boards was held recently to determine the question of providing a joint hospital for the said local board districts.

THE question of a new hospital is being mooted at Paisley. The directors of the infirmary state that they are prepared to consider either an extension is required of existing buildings or a new and enlarged institution.

MR. HOPE MORLEY, hon. treasurer of the Royal Hospital for Diseases of the Chest, City Road, has sent £100 as a "New Year's gift" for that institution. A further sum of £500 has been sent to this hospital by an ex-governor.

LAST month £3,000 still remained to be raised to meet the deficiency on the fabric of the building of the Southport Infirmary. Towards the sum of £17,400 required for completing the buildings, only £14,000 has been subscribed so far.

THE annual meeting of the subscribers of the Alexandra Bromo-Iodine Hospital, Woodhall Spa, was recently held at Lincoln. There have been admitted to this institution 120 patients during the year. Annual subscriptions amounted to £222.

THE Queen has sent gifts of old linen to St. George's Hospital, the London Hospital, and the Cancer Hospital (free), Brompton, and game and linen to Charing Cross Hospital and the Royal Free Hospital, Gray's Inn Road, for the use of the inmates.

ONE of the most valuable additions, which have ever been made to the Agassiz Museum of Comparative Zoology at Cambridge, is a collection of 3,000 birds. The collection was made by Mr. W. E. D. Scott, from the UnitedStates and from the West Indies.

DR. LEVI C. LANE, of San Francisco, has presented to the city of San Francisco a large building with two wings, each two stories high, which he has had built for a fully-equipped hospital, to be used as an annex to the Cooper Medical College of that city.

THE nineteenth annual general meeting of the Holmesdale Cottage Hospital reported that the work of this institution has been carried on steadily and efficiently during the past year. The accounts, unfortunately, for the year show a slight decrease of income.

IT is to be hoped that increased subscriptions will soon be coming in for the proposed cottage hospital at Wood Green. Mr. Passmore Edwards' munificent contribution of £2,000 towards this scheme should establish a precedent for further supplies in support of this much needed project.

LORD CADOGAN, in presenting an appeal for the funds of the Chelsea Hospital for Women, states that 600 in-patients and 13,0 0 out-patients are annually relieved by this charity. The convalescent home at St. Leonards-on-Sea in connection with it is doing a work of real value and mportanc e.

THE Loid Lieutenant of Cambridgeshire has given the munificent donation of £1,000 towards the funds of Addenbrooke's Hospital at Cambridge.

THE working men of Swansea are setting a good example to other districts, in the meritorious manner in which they are contributing towards the Swansea Hospital.

THE petition which has been presented to the Local Government Board of Canterbury by the residents of the town urges that the site selected for the proposed infectious diseases hospital is wholly unsuitable. The piece of land selected immediately adjoins the infirmary attached to the Canterbury Union, and has further serious disadvantages.

IN the city of St. Louis, America, it is intended to organise a street railway ambulance, which will run at stated and known hours to convey more quickly than would otherwise be possible, patients to the various hospitals. Possibly the idea might be applied to our Metropolitan railways, where ordinary cab conveyance would not be suitable.

THE kindly thought shown by the family of the late Sir Andrew Clark, might establish a precedent well worth following on many a similar occasion. After his funeral many of the beautiful flowers sent in his memory by friends were sent to the wards of the London Hospital, where after having honoured the dead, they gave great pleasure to the sick whom he lived to help.

THE London and North-Western Railway Company have offered to give a site for the erection of a cottage hospital near the Queen's Park, Crewe, which the company gave to the town in the Jubilee year.

IN view of this object, Mr. F. W. Webb, chief mechanical engineer at Crewe, has contributed £1,000 towards the erection of the buildings, and two further sums of £1,000 each are promised. The trustees of the late Alderman Heath have decided to give £650 to the same project.

### Medical Vacancies and Appointments.

### APPOINTMENTS.

P. E. Bearblock, M.R.C.S.Eng., L.R.C.P.Lond., appointed Assistant House Surgeon to the Royal Albert Hospital, Devonport. S. A. Bull, M.R.C.S., L.R.C.P., appointed Junior House Surgeon to the Westminster Hospital. M. Cameron, M.D. Glasg., appointed Professor of Midwifery at the University of Glasgow. Dr. Crawford appointed Medical Officer for the Carlton District of the Worksop Union. R. Davies, M.B. and C.M.Edin., M.R.C.S.Eng., L.R.C.P.Lond., appointed Resident Surgeon to the Branch Dispensary of the Cheltenham General Hospital. G. S. Farquharson, M.B.Lond.Univ., M.R.C.S.Eng., L.R.C.P.Lond., appointed Sanitary Surveyor of the Board of Trade for the Port of Southampton (jointly with Dr. Grange). W. D. Gimson, M.R.C.S., L.R.C.P., appointed Medical Officer for the Springfield and Boreham District of the Chelmsford Union. L. D. Gover, M.R.C.S.Eng., L.R.C.P.Lond., appointed Resident Assistant to the Wolverhampton and Staffordshire General Hospital, Wolverhampton. F. Grange, M.D.Lond.Univ., D.P.H.Camb., M. R.C.S.Eng., L.R.C.P.Lond., appointed Sanitary Surveyor to the Board of Trade for the Port of Southampton (jointly with Dr. Farq uharson); also Medical Superintendent of Quarantine under the Privy Council. R. G. Hann, M.R.C.S., L.R.C.P., appointed Resident Medical Officer to the Leeds Public Dispensary. R. W. Henry, M.B., B.Ch.Dub., reappointed Junior House Surgeon to the Birmingham and Midland Eye Hospital. C. A. Hill, M.B., B.C., B.A.Cantab, M.R.C.S.Eng., L.R.C.P.Lond.. appointed House Surgeon to St. George's Hospital. W. D. James, M.R.C.S.Eng., L.S.A., appointed Surgeon for Skin Diseases at the Sheffield General Infirmary. C. H. McIlraith, M.A., M.B., C.M.Glasg., appointed Resident Medical Officer to the Hospital for Diseases of the Throat, Golden Square, W. L. Remfry, M.A., M.D.Cantab, M.R.C.P., appointed Assistant Lecturer on Obstetric Medicine, and Assistant Obstetric Physician to St. George's Hospital. S. R. Thompson, M.R.C.S., L.R.C.P.Lond., appointed Medical Officer for the Parish Street Workhouse and No. 1 District of the St. Olave's Union. H. B. Vincent, M.R.C.S.Eng., Z.S.A., appointed Medical Officer of Health for the Mitford and Launditch Rural Sanitary Authority.

### VACANCIES.

#### Resident Medical Officers.

The following vacancies are announced this week, the amount of salary in each case being given after the name of the institution:

Bradford Infirmary. Dispensary Surgeon, doubly qualified. Salary £100 per annum, with board and residence. Applications, stating age and experience, with testimonials, to the Secretary by January 23rd.

Buckinghamshire General Infirmary, Aylesbury. Resident Surgeon and Apothecary, unmarried and duly qualified. Salary £80, increasing £10 per annum to £100, with board, lodging, and washing. Applications and testimonials to Mr. George Fell, Solicitor, Aylesbury, by January 29th.

Central London Ophthalmic Hospital, Gray's Inn Road, W.C. House Surgeon. Applications, with testimonials, to the Secretary by February 5th.

Darlington Hospital and Dispensary. House Surgeon, doubly qualified and unmarried. Salary £100 per annum, with board and lodgings. Applications, with testimonials, to the Honorary Secretaries, 88, Northgate, Darlington, by January 27th.

Liverpool Infirmary for Children, Myrtle Street, Liverpool. House Surgeon. Salary £85 per annum, with board and lodging. Applications and testimonials to be sent by January 22nd.

Wrexham Infirmary and Dispensary, Wrexham. House Surgeon. Salary £80 per annum, with board, furnished rooms, gas, coal, and attendance. Applications to Mr. George Whitehouse, 27, Regent Street, Wrexham, by January 24th.

#### Non-Resident Medical Officers.

Finsbury Dispensary, Brewer Street, Goswell Road, E.C. Physician. Honorarium of £40 per annum. Applications and testimonials to D. W. Williams, Honorary Secretary, by January 27th.

Royal Pimlico Dispensary, Buckingham Palace Road, S.W. Attending Medical Officer; must reside in the district. Applications and testimonials to the Secretary by February 5th.

University College of South Wales and Monmouthshire. Lecturer on Materia Medica and Pharmacy. Stipend £50 a year. Applications to Ivor James, Registrar, by January 27th.

### The Hospital Advertiser.

### BOOKS FOR NURSES.

Demy 8vo., 200 pp., cloth extra, price 3s. 6d.,

**SURGICAL WARD-WORK AND NURSING**: A practical Manual ot Clinical Instruction for Nurses and Students. This book will be found especially useful to beginners, to whom its simple description of elementary principles and treatment in surgery will render it invaluable. By ALEXANDER MILES, M.D. (Edin.), C.M., F.R.C.S.E. Over one hundred illustrations.

LIST OF CHAPTERS.

SECTION I.—ANTISEPTIC SURGERY. Chap. 1. General Principles of Antiseptic Surgery—2. Antiseptic Lotions—3. Antiseptic Powders and Unguents—4. Materials for Dressings—5. Lotion Basins—6. Ward Table and Dressing Tray—7. Antiseptic Dressing—8. A Spray Dressing—9. Management of Surgical operations—10. The Lotion Table—11. Dressings Table—Anæsthetics—12. Preparation of Patient for Operation—13. Treatment after Operation—14. Nursing of Special Cases after Operation.

SECTION II.—THE USE OF REST IN SURGERY. 15. Extension Apparatus—16. Plaster and Starch Cases—17. Appliances for Joint Affections—18. Appliances for Fractured Bones.

SECTION III.—BANDAGING. 19. Bandaging—20. Special Bandages—21. Special Bandages (*continued*)—22. Sspecial Bandages (*continued*).

SECTION IV.—SURGICAL INSTRUMENTS AND APPLIANCES. 23. General Instruments—24. Instruments for Hæmorrhage—25. Knives—26. Saws—27. Bone Instruments—28. Instruments used in Trephining—29. Mouth Instruments, etc.—30. Intestinal Instruments—31. Urethral Instruments—32. Lithotomy and Lithotrity Instruments—33. Laryngeal Instruments—34. Aural, Nasal, and Ophthalmic Instruments—35. Gynæcological Instruments.

"Will furnish much-needed guidance to the student and the nurse in the early stages of their apprenticeship."—*Times.*

"This is a fully illustrated handbook for the use of junior students of medicine and nurses, which should prove of very great value, Dr. Miles being unquestionably an authority on the subject of which he writes."—*Publishers' Circular.*

Just ready, Important New Text-Book of Ophthalmic Nursing.

Crown 8vo., cloth gilt, pp. 200, profusely illustrated, and with many woodcuts, price 3s. 6d., post free,

**OPHTHALMIC NURSING.** By SYDNEY STEPHENSON, M.B., F.R.C.S.E., Surgeon to the Ophthalmic School, Hanwell, W., &c., &c. A complete text-book on this branch of nursing, illustrated by numbers of original figures, and containing a glossary of terms and list and description of instruments used in ophthalmic nursing.

As yet no book has been published dealing with nursing in diseases of the eye. As a text-book, "Ophthalmic Nursing" will be invaluable to nurses desiring to qualify in this particular branch of nursing. The managers of Ophthalmic Institutions are invited to adopt this work as a text-book for their nursing staffs. The book contains a frontispiece, "Vertical Section of the Eye-ball and Eye-lids," and sixty-one other illustrations. It is concisely yet exhaustively written, and its method of treatment and style will render it especially serviceable to the Nursing Profession.

THE LANCET says: "The work now before us gives the result of his experience to those who have charge of Ophthalmic patients, and will prove useful. A good account is given of the proper arrangements and preparations that should be made for operations, with the various modes of bandaging one or both eyes. The work may very advantageously be studied by nurses and clinical clerks, or dressers who are about to enter the Ophthalmic service of a hospital."

NURSING NOTES says: "A book on Ophthalmic Nursing has been a real need which is now well supplied. . . . . Those contemplating going in for Ophthalmic Nursing should study this useful work. . . . . One of the most useful books for nurses we have seen for a long time."

THE OPHTHALMIC REVIEW says: "The author has evidently endeavoured to write in language which will be intelligible to nurses of all grades, and we congratulate him on the clearness with which his statements are expressed. . . . . Chapters II. and III., upon 'The Germ Theory of Disease' and 'Contagion and Infection,' are good, and the latter contains useful rules for the avoidance by nurses and others of the contagion of eye disease."

"Will prove of inestimable value."—*Publishers' Circular.*

Third Edition, enlarged and partly re-written, cloth gilt, crown 8vo., about 200 pp., price 2s. 6d.,

**HOSPITAL SISTERS AND THEIR DUTIES.** By EVA C. E. LÜCKES, Matron to the London Hospital. Every matron, sister, or nurse who aspires to become a sister should read the valuable advice and information contained in this book. The author's experience as matron of London's largest hospital peculiarly qualifies her to speak on this subject.

"We cordially recommend the work to all hospital authorities as well as to the class for whose special benefit it is more immediately intended."—*Daily Chronicle.* "On every page we find evidence of Miss Lückes' thorough acquaintance with all the details of the nursing department of hospital management."—*Lancet.* "It is well worthy of study for those to whom it is addressed, and for all who are interested in the matter of nursing."—*New York Medical Journal.*

London: THE SCIENTIFIC PRESS (Limited), 428, Strand, W.C.

### ACCOUNT BOOKS FOR INSTITUTIONS.

Designed by HENRY C. BURDETT.

Ruled in accordance with the uniform system of accounts advocated by the Metropolitan Hospital Sunday Fund, and prepared for the convenience and assistance of Secretaries who present annual returns for participation in the Hospital Sunday Fund Grants.

These Books are ruled so that the various descriptions of Receipts and Expenditure, &c., may be entered uniformly under the special headings and in the columns prepared for them.

There are TWO SERIES, No. 1 being for the larger Institutions, and No. 2 for Cottage Hospitals and Small Institutions. The sets are sold complete, at a specially reduced price; or each book may be had separately, at the prices set out below:—

### SERIES No. 1.—FOR HOSPITALS AND INSTITUTIONS.

(Any of these Books may be purchased separately; a reduction is made if the complete set is taken.)

1. Analysis Journal. Royal, 350 pp. Divided into seven sections, with parchment tags. Headings as follows:—
   A. Maintenance.
   1. Provisions. 5. Rent.
   2. Surgery & Dispensary. 6. Salaries.
   3. Domestic. 7. Miscellaneous Expenses
   4. Establishment Charges.
   B. Administratation.
   1. Management. 2. Finance.
   (Uniform with Headings in Income and Expenditure Table.) Each Heading is ruled with pages containing 15 columns. Price £3.
2. Cash Book. Foolscap, 300 pp., printed Headings, specially ruled. Price £1.
3. Cash Analysis and Receipt Book. Foolscap, extra width, 300 pp., ruled, with 15 columns, printed Headings identical with Income and Expenditure Table. Price £1 5s.
4. Secretary's Petty Cash Book. 300 pp., foolscap, ruled with 13 columns, printed Headings identical with Income and Expenditure Table. Price £1.
5. List or Register of Annual Subscribers. Double foolscap, 300 pp., ruled with 10 columns, printed Headings for the years 1893-1900 inclusive. Price £1 15s.
6. Alphabetical Register Book. Foolscap, 300 pp., ruled, with date, name and address, and cash colums, cut into alphabetical sections, with totals at end for annual balances. Price £1 6s.
7. Subscription Register. Foolscap, ruled with 8 columns, printed Headings for date when subscriptions become due, name and address, and cash columns for years 1893 to 1898 inclusive. Price £1 1s.
8. Linen Register. Foolscap long quarto, 300 pp., ruled with columns for Stock, Condemned, Remaining, and Issued Linen, also total in Ward and Remarks, also printed lists of Hospital Linen. Price 8s. 6d.
9. Income and Expenditure Table. Basis of uniform system. For yearly balances and statements. Royal. Price 6d. per sheet, or 5s. per dozen.
10. Special Appeal Account Table. Foolscap. Price 3d. per sheet, or 2s. 6d. per dozen.

Price of Set Complete, £10 Net.

### SERIES No. 2.—FOR COTTAGE HOSPITALS AND SMALLER INSTITUTIONS.

1. Cash Analysis, Receipt, and Expenditure Book. Double foolscap, 300 pp., ruled with 18 columns, printed Headings identical with Income and Expenditure Table, specially prepared for Cottage Hospitals and Small Institutions. Price £1 15s.
2. Secretary's Petty Cash Book —300 pp., foolscap, ruled with 13 columns, printed headings identical with Income and Expenditure Table. Price £1.
3. Income and Expenditure Table. Basis of uniform system. For yearly balances and statements. Royal. Price 6d. per sheet, or 5s. per dozen.
4. Linen Register. Foolscap, 300 pp., ruled with columns for Stock, Condemned, Remaining, and Issued Linen, also total in Ward and Remarks, also printed lists of Hospital Linen. Price 8s. 6d.
5. List or Register of Annual Subscribers. Foolscap. 300 pp., ruled with columns for the years 1893-1900 inclusive. Price £1.
6. Special Appeal Account Tables. Foolscap. Price 3d. per sheet, or 2s. 6d. per dozen.

Price of Set Complete, £4 Net.

The books are all uniformly bound in half basil, with gilt letterings, and are ruled on best paper, and in every way prepared for practical use at the offices of Institutions.

### ACCOUNTS, AUDIT, AND TENDERS; the Uniform System of,

For Hospitals and Institutions, with Certain Checks upon Expenditure. Also the Index of Classification. Compiled by a Committee of Hospital Secretaries, and adopted by a General Meeting of the same, 18th January, 1892. And certain Tender and other Forms for securing Economy. By HENRY C. BURDETT. Demy 8vo, cloth, 6s.

This is a hand-book to the account books, expla ning their nature and uses, the method of keeping them, and the general principles upon which they were designed.

All having to do with hospital accounts will find much valuable information in this work.

Secretaries making up returns for participation in the Donations of the Metropolitan Hospital Sunday Fund will find it invaluable. It will be of special interest, also, to the officials of all Institutions—Treasurers, Secretaries, and Auditors—as it expounds practically the Uniform System, which is now being widely adopted as a means of saving labour and of bringing all systems of accounts into uniformity.

"This useful and practical little book. No one interested in the hospital question can treat with indifference anything which falls from Mr. Burdett. . . . A uniform system of keeping their accounts. . . . Would be a boon to hospitals generally."—*British Medical Journal.*

London: The Scientific Press (Limited), 428, Strand, W.C.

